# Actinomycete Potential as Biocontrol Agent of Phytopathogenic Fungi: Mechanisms, Source, and Applications

**DOI:** 10.3390/plants11233201

**Published:** 2022-11-23

**Authors:** Juan A. Torres-Rodriguez, Juan J. Reyes-Pérez, Evangelina E. Quiñones-Aguilar, Luis G. Hernandez-Montiel

**Affiliations:** 1 Nanotechnology and Microbial Biocontrol Group, Centro de Investigaciones Biológicas del Noroeste, Av. Politécnico Nacional 195, Col. Playa Palo de Santa Rita Sur, La Paz 23090, Mexico; 2 Facultad de Ciencias Pecuarias, Universidad Técnica Estatal de Quevedo, Av. Quito km 1.5 vía a Santo Domingo, Quevedo 120501, Ecuador; 3 Centro de Investigaciones y Asistencia en Tecnología y Diseño del Estado de Jalisco, Camino Arenero, El Bajío del Arenal, Guadalajara 45019, Mexico

**Keywords:** antifungal activity, marine, saline, wetland, post-harvest

## Abstract

Synthetic fungicides have been the main control of phytopathogenic fungi. However, they cause harm to humans, animals, and the environment, as well as generating resistance in phytopathogenic fungi. In the last few decades, the use of microorganisms as biocontrol agents of phytopathogenic fungi has been an alternative to synthetic fungicide application. Actinomycetes isolated from terrestrial, marine, wetland, saline, and endophyte environments have been used for phytopathogenic fungus biocontrol. At present, there is a need for searching new secondary compounds and metabolites of different isolation sources of actinomycetes; however, little information is available on those isolated from other environments as biocontrol agents in agriculture. Therefore, the objective of this review is to compare the antifungal activity and the main mechanisms of action in actinomycetes isolated from different environments and to describe recent achievements of their application in agriculture. Although actinomycetes have potential as biocontrol agents of phytopathogenic fungi, few studies of actinomycetes are available of those from marine, saline, and wetland environments, which have equal or greater potential as biocontrol agents than isolates of actinomycetes from terrestrial environments.

## 1. Introduction

One of the key problems in agriculture is the damage caused by phytopathogenic fungi [1]. Conventional methods to control phytopathogenic fungi have been carried out by using synthetic fungicides; however, their application causes resistance in microorganisms and harm to human, animal, and environmental health [2]. With the objective of achieving food production efficiency from an ecological and economic points of view, the search for an alternative to decrease the use of synthetic fungicides in agriculture is a global priority [3].

In recent years, the use of actinomycetes as a biocontrol agent on phytopathogenic fungi has been an alternative to the application of synthetic fungicides [4]. Actinomycetes are Gram-positive bacteria found in different habitats, humidity, pH, and temperature [5]. Actinomycetes have been isolated from different environments, such as terrestrial, marine, hypersaline, wetlands, and plant endophytes, among others [6,7].

The main antagonistic mechanisms of actinomycetes to control phytopathogenic fungi are competence for space and nutrients [8], antibiotics [9], siderophores [10], lytic enzymes [11], volatile organic compounds (VOCs) [12], and host resistance induction [13]. Additionally, actinomycetes promote plant growth and development through the synthesis of phytohormones, atmospheric nitrogen fixation, and mineral solubilization, among others [14]. Several studies of actinomycetes have been reported; however, few studies have focused on actinomycetes, isolated from different environments, used as biocontrol agents due to their effect in agriculture and antagonistic mechanisms to phytopathogenic fungi.

## 2. General Characteristics of Actinomycetes

Actinomycetes form vegetative or aerial mycelia and are capable of reproducing by binary fission [15]. In vitro culture and the natural environment have a typical smell of humid soil because of the production of two geosmin and 2-methylisoborneol volatile organic compounds [16]. Spore production is a result of nutrient depletion, allowing actinomycetes to remain latent until they find favorable conditions for growth [14]. Additionally, filamentous and sporulating natures allow them to compete more efficiently against other organisms found in the rhizosphere [17]. Their cell wall is a rigid structure formed by complex compounds, such as peptidoglycan, teichoic and teichuronic acids, and polysaccharides. Actinomycetes also have a high guanine and cytosine content in DNA [18].

## 3. Actinomycetes as Biocontrol Agents

The importance of the use of actinomycetes as biocontrol agents is explained by inherent positive characteristics: (1) they are not harmful to human and animal health; (2) they are not toxic to plants; (3) they improve plant yield; and (4) they decrease the use of synthetic fungicides [19,20]. Among the different genera, *Streptomyces* has been investigated extensively because it is easy to isolate [21]. Actinomycetes have a slower growth than bacteria. Thus, growth improvement techniques should be applied to obtain desirable actinomycetes in culture media. These techniques are based on selective isolation media and the pretreatment of samples, such as: soil with calcium carbonate, both by drying and heating, wet, and chemical pretreatments, among others [22]. One of the ways to stimulate actinomycete populations in soil is by adding biostimulants and organic fertilizers, such as compost and vermicompost. *S. sampsonii* and *S. flavovariabilis* isolates from soil amended with vermicompost showed the highest antagonistic activity towards *Rhizoctonia solani, Alternaria tenuissima*, *Aspergillus niger*, and *Penicillium expansum* [23]. In addition, soil amended with *Brassica napus* and *Brassica rapa* leaf residues promoted the increase in actinomycete populations in the soil. The increase in the actinomycete population showed a strong correlation with the suppression of the *R. solani* wilt disease [24]. Different actinomycetes have been studied as biocontrol agents on phytopathogenic fungi and as mechanisms of action (Table 1).

## 4. Main Actinomycete Antagonistic Mechanisms to Phytopathogenic Fungi

**Figure 1:**
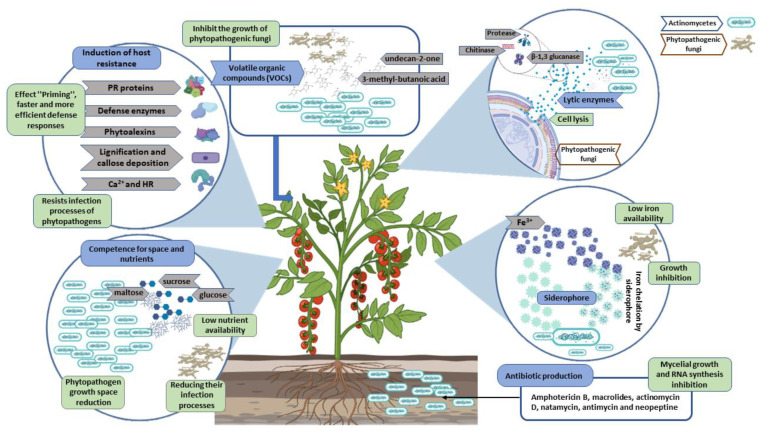
Main actinomycete antagonistic mechanisms to phytopathogenic fungi.

Biocontrol agents use a combination of several antagonistic mechanisms of action to control phytopathogenic fungi [30]. The main antagonistic mechanisms of actinomycetes are their competence for space and nutrients, antibiotics, siderophores, lytic enzymes, and induction of host resistance, among others [1,13,19] (Figure 1). Understanding the mechanisms of action of biocontrol agents is essential in order to improve their viability and increase their potential [31].

### 4.1. Competence for Space and Nutrients

Competition is an indirect mechanism of actinomycetes for the growth inhibition of phytopathogenic fungi [8]. Competence between two or more microorganisms begins for the same carbon source (carbohydrates such as sucrose, glucose, maltose, and fructose) or space for their growth [32,33]. The ecological plasticity and fast growth of antagonistic microorganisms allow them to assimilate the available nutrients in the host at a greater amount than phytopathogenic fungi; thus, the spore germination stage and infection processes to the host are reduced [34]. Competence is also an effective biocontrol mechanism when the antagonist is found in sufficient volumes and assimilates nutrients faster and in greater quantity than phytopathogenic fungi [30].

### 4.2. Antibiotic Production

Actinomycetes produce secondary metabolites with antifungal properties [1]. Approximately 80% of antibiotics, such as streptomycin, spectinomycin, neomycin, tetracycline, erythromycin, and nystatin, are produced by actinomycetes [35]. 

Furthermore, many metabolites have been discovered with antimicrobial properties similar to phytopathogenic fungi, such as amphotericin B, macrolides, actinomycin D, natamycin, antimycin, and neopeptine [36,37,38]. Macrolides are a group of antibiotics produced by actinomycetes that inhibit fungus protein synthesis [39]. Amphotericin B joins selectively to ergosterol in the fungal cell membrane, producing changes in permeability and inducing cell lysis [40]. Moreover, actinomycin D production by *Streptomyces* sp. strains limit microbial growth and RNA synthesis [9]. Antimycin inhibits the mitochondrial electron transport chain between cytochromes *b* and *c* [41]. Natamycin blocks fungal growth when it joins to the ergosterol of the fungus cell membrane [42]. Neopeptine is an inhibitor of the microbial cell wall biosynthesis at the enzymatic level [37]. Another important process that involves antibiotic production is symbiosis between actinomycetes and plants, as the antibiotic protects the plant from phytopathogenic fungi, and the plant exudates allow actinomycete development [43].

### 4.3. Siderophore Production

Siderophores are molecules that perform sequestration on low-molecular weight irons (500–1000 Da) and link with Fe^3+^ ions to be transported to the cell and secreted in response to low Fe^3+^ availability [44]. Siderophores are classified as: phenolate, catecholate, hydroxamate, and carboxylate; some have a group mix (mixed types) [45]. Siderophore production has been demonstrated by *Streptomyces* strains that produce hydroxamate-type siderophores known as deferoxamine [20]. Moreover, heterobactins are catecholate-hydroximate mixed-type siderophores that have been found in *Rhodococcus erythropolis* [46], and albisporachelin is a hydroxamate-type siderophore produced by *Amycolatopsis albispora* [47]. A sufficient amount of siderophore production by biocontrol agents limits Fe^3+^ availability for phytopathogenic fungi. Thus, growth and virulence are limited because microorganisms without iron in their environment cannot perform vital processes, such as synthesis and repair of nucleic acids, respiration, photosynthetic transport, and nitrate reduction or free radical detoxification [48].

### 4.4. Lytic Enzyme Production

Actinomycetes produce lytic enzymes, such as chitinase, β-1,3-glucanase, and protease that degrade the fungal cell wall [26] and cause loss of membrane integrity, set intracellular material free, and cell death [1]. The fungus cell wall is responsible for a cell’s physical integrity, formed by chitin, β-1,3-glucan, and protein [49]. The β-1,3 glucanase hydrolyze β-D-glycosidic bonds of β-1,3 glucan, and chitinases hydrolyze chitin β-1,4 N-acetyl-β-D-glycosamide bonds, breaking fungal cell walls [31,50]. Proteases hydrolyze proteins, specifically mannoproteins, make up the phytopathogenic fungi cell wall [51].

### 4.5. Volatile Organic Compounds (VOCs)

Volatile organic compounds (VOCs) are low molecular weight compounds that evaporate easily at a normal temperature and pressure, which gives them the ability to diffuse through the atmosphere and soil [52]. Most VOCs are lipid-soluble and thus have low water solubility. These organic compounds travel great distances in structurally heterogeneous environments, as well as in solid, liquid, or gaseous compounds [31]. VOCs produced by actinomycetes inhibit the growth of phytopathogenic fungi, promote plant growth, possess nematocidal activity, and induce systemic resistance in plants [11,12]. They inhibit the mycelia, causing swelling, conidia collapse, and structural alterations in the fungal cell wall [53]. The *Streptomyces* species produces 2-ethyl-5-methylpyrazine and dimethyl disulfide that inhibit mycelial growth and spore germination [12]. VOCs such as S-methyl ethanethioate, 1,2-dimethyldisulfane, 2-methyl propanoic acid, acetic acid, 3-methyl-butanoic acid, undecan-2-one, nonan-2-one, and 2-isopropyl-5-methylcyclohexan-1-ol have been reported from the actinomycetes *Nocardiopsis* sp., which inhibit mycelial growth of fungi [54].

### 4.6. Induction of Host Resistance

Induced resistance in plants is activated by antagonist actinomycetes that cause a defense response in the host through several chemical or biochemical reactions [13]. Systemic acquired resistance (SAR) and induced systemic resistance (ISR) are two forms of induced resistance, characterized based on signaling pathways [30]. SAR stimulates a rapid response in the phytopathogens and actinomycetes, stimulating a special ISR state called “priming”, for faster and stronger defense responses [55,56]. Actinomycetes are capable of inducing defense responses in plants through the overproduction of: (1) enzymes related to defense, which strengthen the cell wall structure, avoiding the entrance of phytopathogenic fungi, their colonization toward the plant, and catalyzing phenolic compound oxidation to quinones that are toxic for fungi [29]; (2) proteins (PR) related to pathogenesis, such as chitinase hydrolytic enzymes, and β-1,3-glucanase that break the phytopathogenic fungi cell wall structure [57]; (3) phytoalexins, which are toxic for phytopathogenic fungi, inhibit germ tube elongation and growth, decrease mycelial growth and limit glucose absorption [30,58]; (4) lignification promotion that contributes to plant cell wall hardening [59]; and (5) callus formation induction that isolates stress (biotic and abiotic) in the tissue, locally, by depositing a physical barrier [56,60].

## 5. Actinomycete Isolation from Different Environments

Actinomycetes have been isolated from different environments, such as terrestrial, marine, hypersaline, wetland, as well as plant endophytes, among others. Marine environments cover more than 67% of terrestrial surface, and only 1% of the microorganisms have been studied [61]. Marine actinomycetes living in extreme environmental conditions are ideal for the synthesis of new secondary metabolites because of their adaptation to reproduce, grow, and feed [5]. Endophyte actinomycetes are microorganisms that inhabit plant tissues during the totality or part of the life cycle and do not cause negative effects in the host [17]. Additionally, molecules produce functions as growth promoter metabolites, antimicrobials to phytopathogenic fungi, improve gene expression of plant defense that codify enzymes, such as phenylalanine ammonia-lyase (PAL), and improve nutrient absorption [62]. Wetlands are biologically important ecosystems that provide habitat, food, and spawning areas for a number of plants and animals [7]. Hypersaline environments are extreme habitats with high concentrations of salt, alkalinity, and low oxygen. Actinomycetes have been isolated from different hypersaline environments, such as salt lakes, salt flats, salt mines, and brine wells; however, these environments remain unexplored [6]. Compounds and secondary metabolites of terrestrial microorganisms have been studied extensively, hence the importance of searching for new isolation sources for actinomycetes [33].

## 6. Antifungal In Vitro Activity of Actinomycetes Isolated in Different Environments

**Figure 2:**
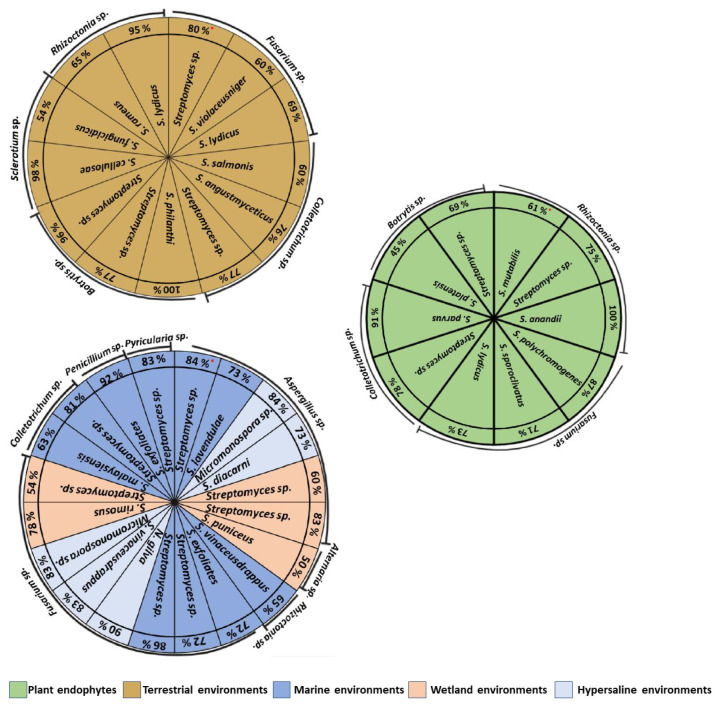
Antifungal activity (% growth inhibition *) in vitro of actinomycetes isolated from different environments [1,3,4,6,10,19,20,21,53,59,60,64,65,66,67,68,69,70,71,72,73,74,75,76,77,78,79,80,81,82,83,84,85].

The *Streptomyces*, *Micromonospora*, and *Nocardiopsis* species are within the main actinomycetes that have been studied for their antifungal in vitro activity [63,64] and isolated from different environments, such as terrestrial, marine, saline, and wetland (Figure 2).

Actinomycetes of terrestrial origin, such as *Streptomyces* sp., have demonstrated to reduce the mycelial growth of *R. bataticola* by 65.3% [10]. Similar results were obtained for *Streptomyces* sp. isolated from a terrestrial environment, reducing the mycelial growth of *Botrytis cinerea* by 77% [59]. The antifungal activity of *Streptomyces* sp. VOCs of terrestrial origin inhibited the mycelial growth of *F. solani* by 69% [53]. In addition, in another investigation, *Streptomyces* sp. VOCs reduced the mycelial growth of *C. acutatum* by 77% [64]. Endophytic actinomycetes from marine and wetland environments have also inhibited the growth of phytopathogenic fungi under in vitro conditions. A study of *S. polychromogenes* endophytes from date palm roots inhibited the mycelial growth of *F. solani*; the in vitro antifungal activity was associated with the production of lytic enzymes that degrade the cell wall [1]. A *Streptomyces* sp. Extract of marine origin containing oligomycin A inhibited the growth of *Pyricularia oryzae* hyphae by 83%, which damaged the fungal membrane, inhibited conidial germination and appressoria formation [65]. *Streptomyces* spp. From marine environments have also inhibited the growth of *Penicillum digitatum*, *A. niger* and *F. solani* by 92, 73, and 72%, respectively [66]. Actinomycetes from marine environments, such as *Streptomyces* sp. And *N. lucentensis*, inhibited the mycelial growth of *F. solani* by 72 and 68%, respectively, and *Streptomyces* sp. showed no significant differences with the synthetic fungicide [67].

## 7. Antifungal Activity of Actinomycetes In Vivo Isolated from Different Environments

**Figure 3:**
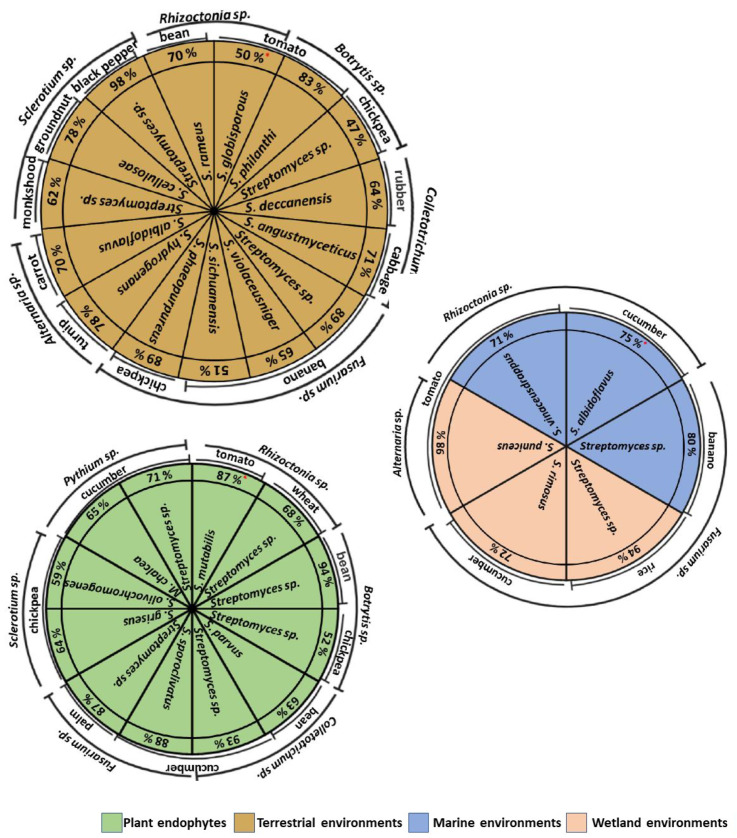
Antifungal activity (% growth inhibition *) of actinomycetes in vivo isolated from different environments [1,4,10,21,25,29,59,60,63,69,70,71,73,74,77,78,80,83,85,87,88,89,90,91,92,93,94,95,96,97].

The diseases transmitted by soil phytopathogenic fungi are difficult to control with synthetic fungicides [21]. Plant diseases cause a yield loss of 50%, particularly in developing countries [86]. The antifungal activity of actinomycetes isolated from different environmental conditions has been demonstrated in vitro conditions. However, research in actinomycetes as biocontrol agents in vivo conditions has been limited to the study of terrestrial actinomycete isolates (Figure 3) due to difficulties in sampling and culturing microorganisms of marine, saline, and wetland environments, among others [5]. Nevertheless, interest still exists in finding more efficient strains that differ considerably with respect to their biocontrol efficiency [19].

*Streptomyces* species from terrestrial environments have been shown to significantly reduce the incidence of *B. cinerea* disease on chickpea plants by 47%, compared to the control, and induce resistance in the host plant through antioxidant enzymes and phenolic compounds [59]. The antifungal activity of the *S. sichuanensis* strain from terrestrial environments towards *F. oxysporum* was associated with siderophore production and whose extracts induced apoptosis of phytopathogen cells. In the greenhouse experiment, the *S. sichuanensis* strain significantly inhibited *F. oxysporum* infection in roots and bulbs of banana seedlings and reduced the disease index by 51% [25].

Moreover, the actinomycete endophytes of date palms decreased the sudden decline syndrome (SDS) disease, caused by *F. solani*, by 86% under greenhouse conditions; these effects are related to the production of antifungal metabolites of the *S. coeruleoprunus* strain [1]. Studies of *Streptomyces* sp. extracts from marine environments have shown that the disease index of *F. oxyspoum* significantly decreased by 80%. This effect could have been associated with secondary metabolites causing the loss of osmotic balance, cell membrane rupture and leakage of cellular components of *F. oxyspoum* [85]. Similarly, the application of *S. vinaceusdrappus* from the marine environment on tomato plants showed a disease reduction (71%) of root rot caused by *R. solani* compared to the untreated control [73]. In detached tomato leaves, co-inoculation of *A. solani* with *S. puniceus* extract from wetland environments reduced the disease by 98%, relative to the control, due to the presence of antifungal metabolites, such as *Alteramide A* [77]. These investigations confirm the potential of actinomycetes isolated from different environments, not only terrestrial, in plant disease management.

## 8. Antifungal Activity of Actinomycetes Isolated in Different Environments in Postharvest Fruit

**Figure 4:**
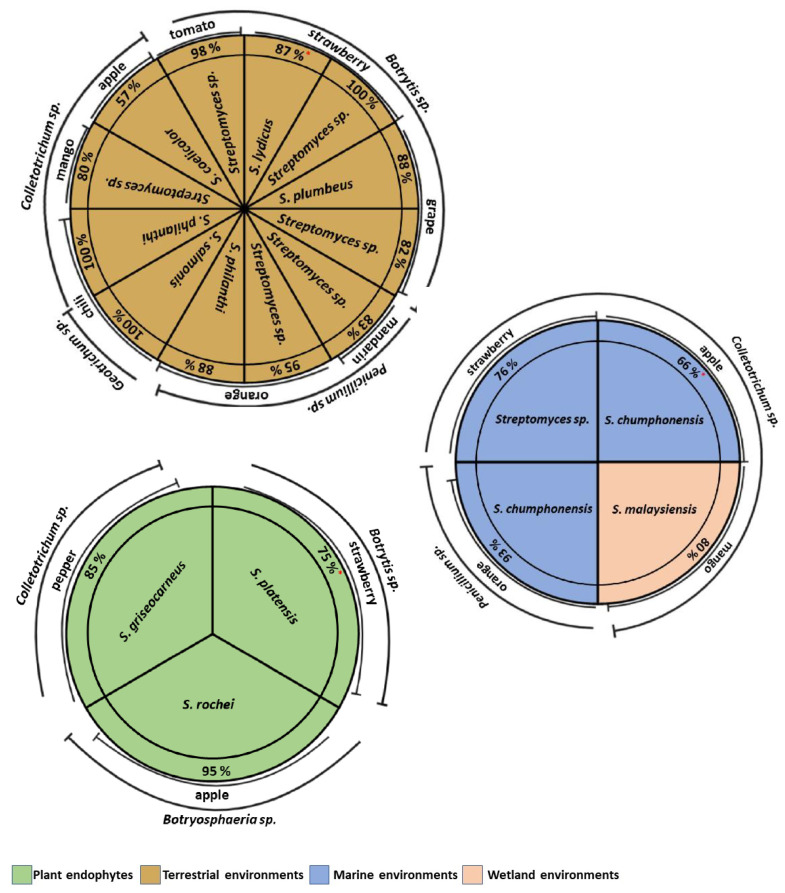
Antifungal activity (% growth inhibition *) of actinomycetes in post-harvest isolated from different environments [3,19,20,53,64,68,84,102,103,104,105,106,107,108,109].

The main losses in post-harvest fruit are caused by phytopathogenic fungi, which represent more than 50% of agricultural production [98]. In post-harvest fruit management, antagonists are subjected to changes in pH, temperature, and humidity because in these conditions the efficiency of biocontrol agents can be affected [99]. Actinomycetes from marine, saline, hypersaline, and wetland environments are subjected to extreme environmental conditions that allow them to adapt to the changes in temperature, pH, and humidity that occur post-harvest [19,100]. However, in most of the studies of actinomycetes and biocontrol of phytopathogenic fungi in post-harvest fruit, the isolates provided are from terrestrial environments (Figure 4). More studies should be performed with these microorganisms isolated from different environments.

In the post-harvest trial on strawberries inoculated with *B. cinerea*, VOCs from *Streptomyces* sp. isolated from terrestrial environment inhibited the development of gray mold symptoms on fruit by more than 87% compared to untreated control strawberries. In addition, *B. cinerea* conidia showed symptoms of swelling and crumbling and the fungal mycelium showed structural alterations [53]. Moreover, incubation of apples infected with *C. acutatum* in semi-closed boxes with *Streptomyces* sp. strains showed that the VOCs produced by *Streptomyces sp*. reduced the rotting areas of the apples by 66% in relation to the control treatment [64].

Marine actinomycetes, such as *S. chumphonensis*, reduced citrus green mold disease caused by *P. digitatum* by 93%. The authors suggest that this effect may be related to the production of antimicrobial substances [101]. Furthermore, *Streptomyces* sp. species from marine environments and their metabolites showed high efficacy in the control of *C. fragariae* in strawberry fruit, reducing the severity of anthracnose disease by 76%, in addition, fruit hardness and color were maintained [80].

## 9. Commercial Products Based on Actinomycetes

The main problem in obtaining commercial products based on microorganisms is that their biocontrol capacity is different in in vitro trials and field experiments. In addition, developing a commercial microorganism product is a complex, time-consuming, costly and interactive process. The success of a biocontrol agent is its formulation, which must include a specific concentration of the new microorganisms and a set of other inert ingredients to produce a commercial product for its use in field conditions, and must show repeated positive results, reasonable prices and easy handling [17]. The efficiency of these biocontrol agents is affected by environmental factors, such as temperature, humidity, precipitation, among other abiotic aspects which synthetic fungicides have overcome [33]. 

The factors outlined above all make the transfer of an effective biocontrol agent under controlled laboratory conditions to a commercially available product for application under field conditions difficult. Although the use of microorganisms as a biocontrol agent is a current option to reduce synthetic fungicides, the ratio of actinomycetes registered as biocontrol agents for commercial availability is still low [110]. From the commercial products based on actinomycetes, Mycostop is the only product registered in Canada, the European Union, and the United States of America (Table 2). Overall, an open field for the industry is envisaged for actinomycete-based products in agriculture.

## 10. Conclusions

Actinomycetes are an option to control phytopathogenic fungi in agriculture and their application reduces the use of synthetic fungicides. Marine, saline, and wetland environments are important sources for actinomycete isolation and in the discovery of new compounds and secondary metabolites. Biocontrol studies have focused on isolates of actinomycetes from terrestrial environments. Nevertheless, actinomycetes from marine, saline, and wetland environments have equal or greater antifungal activity than those from terrestrial environments.

## Figures and Tables

**Table 1:** Antagonistic mechanisms of actinomycetes for the control of phytopathogenic fungi.

Actinomycete	Phytopathogen	Host	In Vivo Inhibition	Antagonistic Mechanisms	Reference
*Streptomyces* sp.	*Colletotrichum fragariae*	Strawberry	100%	Secondary metabolites	[19]
*S. sampsonii*	*Sclerotinia sclerotiorum*	Green bean	100%	Secondary metabolites	[11]
*Streptomyces* sp.	*Ralstonia solanacearum*	Tomato	97%	Induction of host resistance	[13]
*S. sichuanensis*	*Fusarium oxysporum*	Banana	51%	Siderophores	[25]
*Amycolatopsis* sp.	*F. graminearum*	Maize	79%	Lytic enzyme	[26]
*Arthrobacter humicola*	*A. alternata*	Tomato	31%	Secondary metabolites	[27]
*Nocardiopsis dassonvillei*	*Bipolaris sorokiniana*	Wheat	72%	Siderophores and lytic enzyme	[28]
*S. rameus*	*R. bataticola*	Bean	70%	Siderophores and lytic enzyme	[10]
*S. globisporous*	*R. solani*	Tomato	50%	Induction of host resistance	[29]

**Table 2:** Commercial products based in actinomycetes.

Commercial Product	Actinomycete	Registered Countries	Phytopathogen Species/Target Disease	Main Effects	Reference
Mycostop	*S. griseovirids*	Canada, UE countries, and USA	*Alternaria, R. solani, Fusarium, Botrytis, Phytophthora*, and *Pythium*	Space and nutrient competence and produces polyenic antibiotics	[111]
Actinovate	*S. lydicus*	Canada and USA	*Pythium, Fusarium, Phytophthora, Rhizoctonia,* and *Verticillium,* powdery and downy mildew, and *Botrytis, Alternaria, Geotrichum,* and *Sclerotinia*	Induces resistance in plants and produces extracellular chitinases	[112]
Mycocide KIBC	*S. colombiensis*	South Korea	Powdery mildews, grey mold, and brown patch	Produces enzymes and antibiotics	[113]
Safegrow KIBC	*S. kasugaensis*	South Korea	Sheath blight and large patch	Produces enzymes and antibiotics	[113]
Kasugamycin, Kasumin	*S. kasugaensis*	Ukraine	Leaf spot, scab, and root rot	Inhibit protein biosynthesis	[114]
Agrimycin, Paushak, Cuprimicin 17, Astrepto 17	*S. griseus*	India, USA, New Zealand, China, Ukraine and Canada	Bacterial rots, *Xanthomona*, and *Pseudomonas*	Inhibit protein biosynthesis	[112]
Polyoxorim (Endorse, Polyoxin Z and Stopit)	*S. cacaoi* var. *asoensis*	UE countries	*Sphaerotheca*, powdery mildews, *Botrytis*, *Sclerotium*, *Corynespora*, *Cochliobolus*, *Alternaria*, sheath blight, and *Helminthosporium*	Inhibit cell wall biosynthesis and causes abnormal spore germ tube swelling and hypha points	[115]
Validacin, Valimun, Dantotsupadanvalida, Mycin Hustler, Valida	*S. hygroscopicus*	-	*Rhizoctonia*	Inhibit trehalase in *Rhizoctonia*	[116]

## Data Availability

Not applicable.
